# Quality of Life in Menopausal Women: A Brazilian Portuguese Version of the Cervantes Scale

**DOI:** 10.1100/2012/620519

**Published:** 2012-03-12

**Authors:** José E. M. Lima, Santiago Palacios, Maria C. O. Wender

**Affiliations:** ^1^Post-Graduate Program in Medicine, Universidade Federal do Rio Grande do Sul, 90040-060 Porto Alegre, Brazil; ^2^Faculdade de Medicina, Universidade de Passo Fundo, 99052-900 Passo Fundo, Brazil; ^3^Menopause Division, Instituto Palacios, 28009 Madrid, Spain; ^4^Obstetrics and Gynecology Department, Universidade Federal do Rio Grande do Sul; Hospital de Clínicas de Porto Alegre, 90035-903 Porto Alegre, Brazil

## Abstract

We present the translation, cultural adaptation and validation of the Cervantes Scale to Brazilian Portuguese. The Cervantes Scale (CS) was originally described in Spanish, and is a tool to measure health-related quality of life in perimenopausal and menopausal women. A cross-sectional study was carried out with 180 women aged 45 to 64 years. In addition to the CS, the following questionnaires were applied: Women's Health Questionnaire (WHQ) and abbreviated version of the World Health Organization's Quality of Life Questionnaire (Abbreviated WHOQOL-bref). In conclusion, the Brazilian Portuguese version of the CS is easy to apply and understand. The evaluation of its psychometric properties was satisfactory, and it can be applied to assess health-related QoL in Brazilian perimenopausal and menopausal women.

## 1. Background

Climacteric symptoms are known to impair quality of life (QoL) [1]. The climacteric syndrome is associated with menstrual irregularities, hot flashes, sweating, palpitations, sleep disturbance, irritability, lethargy, depressed mood, forgetfulness, decreased libido, vulvovaginal dryness, dyspareunia, and urinary symptoms [2]. In addition, during this period, gradual changes occur in bone metabolism, resulting in increased risk for osteoporotic fractures. The incidence of cardiovascular disease also increases significantly after menopause [1, 3].

Over the last two decades, the importance of QoL as a health parameter has grown consistently, and a range of scales has been proposed to evaluate specific population groups [4–6]. Among them, the Cervantes Scale (CS) was developed and validated in a representative sample of Spanish pre-, peri-, and menopausal women [7, 8]. This self-administered questionnaire evaluates four domains: health and menopause, sexuality, couple relationship, and psychological aspects.

The purpose of this study was the translation, cultural adaptation, and validation of the CS in Brazilian Portuguese.

## 2. Methods

This study was conducted in two stages. The first stage included the translation and cultural adaptation of the scale [9], including a pilot study that assessed the degree of understanding of the Brazilian Portuguese version of the Cervantes Scale (BP/CS) by the target population. In the second stage, the BP/CS was validated through statistical analysis of its psychometric properties: internal consistency, intraobserver reliability (test-retest with a two- to four-week interval between applications in women without any major changes in their health status), and validity, based on the comparison of the CS with the Women's Health Questionnaire (WHQ) [4] and the World Health Organization's Quality of Life Questionnaire (WHOQoL-bref) [10], which had been previously validated for BP. A question was added at the end of the BP/CS (“Were there any words that you did not understand?”) to detect opportunities of improvement. The translation followed World Health Organization (WHO) recommendations [9]. Cultural equivalence was established if all the questions were understood by at least 85% of the subjects [11].

Statistical analysis was performed using SPSS 15.0 for Windows. Continuous variables were expressed as mean ± standard deviation and categorical variables were expressed as absolute and relative frequency. Internal consistency was assessed using Cronbach's coefficient. Reliability was assessed using the intraclass correlation coefficient. Correlations between the global BP/CS score, BP/CS domain scores, and BP/CS subdomain scores with the WHQ and the WHOQoL-bref were evaluated using Pearson's correlation coefficient. The associations between global BP/SC, domain, and subdomain scores with sociodemographic, clinical, and behavioral variables were assessed using Student's *t* test and analysis of variance (ANOVA).

Between August 2007 and November 2008, women were consecutively selected for this cross-sectional study from three university clinics and one private clinic in the same city, in southern Brazil. The sample size was calculated as 179 participants using the WinPepi software for an intraclass correlation coefficient of at least 0.7, with precision of 0.15 and significance of 5%. The study was approved by the University's Research Ethics Committee (protocol 002/2007), and all participants signed an informed consent form. A form was initially filled for collection of sociodemographic, clinical, and behavioral data. Then, the questionnaires were applied in the following order: BP/CS, WHQ, and WHOQoL-bref.

Menopause was defined as absence of spontaneous menstrual periods in the past 12 months in women not submitted to bilateral oophorectomy or hysterectomy, who were older than 50 years at the time of the survey. Exclusion criteria were illiteracy or significant visual disability that prevented reading of the questionnaire, life-threatening diseases or clinical instability, and use of antidepressants.

The questionnaires were considered as invalid if three (10% of scale) or more questions were left unanswered. In the presence of one or two unanswered questions, the total score or the score of the area was obtained by multiplying the result by a correction factor [12]. Data were entered into an Excel spreadsheet and revised for missing or duplicated information.

## 3. Results

### 3.1. Translation and Cultural Adaptation

Ten climacteric women (patients from the university clinic and from the private clinic) participated in the pilot study. There were no difficulties with the BP/SC. An expert committee comprising three gynecologists, one psychiatrist, and one general practitioner was also satisfied with the BP/CS.

### 3.2. Sociodemographic, Clinical, Behavioral Features

Table 1 describes the sociodemographic characteristics of 180 women included in the study: 123 (68.3%) from the university outpatient clinic and 57 (31.7%) from the private clinic. Age ranged from 45 to 64 years. One hundred and eleven (61.7%) women had never received hormone therapy (HT), 23 (13.3%) had started and discontinued HT, and 46 (25.6%; 47.4% of the postmenopausal group) were on HT. Natural menopause was observed in 47 (26.1%) women. Mean age at spontaneous menopause was 48.1 ± 4.1 years; 40 (22.2%) women had surgical menopause (hysterectomy). Considering the cutoff point of age 50 years, 13 (7.2%) women were younger and 27 (15.0%) were older than 50 years. Twenty three (12.8%) were not able to report when menopause occurred, and 70 (38.9%) had not yet experienced menopause. Climacteric symptoms were observed in 153 (85.0%) women.

## 4. Evaluation of Psychometric Proprieties

Cronbach's alpha for the global CS score was 0.83. For each domain, Cronbach's alpha was 0.81 for menopause and health, 0.84 for the psychological domain, 0.79 for sexuality, and 0.73 for couple relationship. Concerning subdomains, Cronbach's alpha was 0.85 for vasomotor symptoms, 0.62 for health, and 0.54 for aging.  Table 2 shows the correlations between global CS score and domain and subdomain scores.

We evaluated intraobserver reliability by retesting 66 (36.6%) women. The intraclass correlation coefficient for the global scale was *r* = 0.94, 95% CI: 0.89–0.96 (*P* < 0.001). The correlation for the different domains was menopause and health, *r* = 0.92, psychological, *r* = 0.88, sexuality, *r* = 0.88, and couple relationship, *r* = 0.89. For the three subdomains, the correlation coefficients were vasomotor symptoms, *r* = 0.90, health, *r* = 0.92, and aging, *r* = 0.90 (*P* < 0.001).

Pearson's correlation coefficient for the comparison between the BP/CS and the WHQ and WHOQoL-bref was *r* = 0.79 and *r* = −0.71, respectively (*P* < 0.001).  Table 3 shows the correlation between BP/CS scales and subscales with the WHQ and the WHOQoL-bref. Discriminant validity was observed for schooling, family income, presence or absence of vasomotor symptoms, and frequency of physical activity.

Tables 4 and 5 show the comparison of BP/CS scores in patients with different levels of schooling and income, presence/absence of hot flushes, and different levels of physical activity, respectively. No discriminant validity was observed for the age subgroups (45 to 54 years and 55 to 64 years) and climacteric period (premenopausal and menopausal women with and without hormone therapy).

## 5. Discussion

In the present study, the CS was translated into Brazilian Portuguese and culturally validated. The time required for completion of the BP/SC was 6 to 8 minutes, which is equivalent to that reported for the original Spanish version [7]. The internal consistency observed for the BP/CS was also similar of that of the original CS (>0.7).

Criterion validity was assessed via the correlation of the BP/CS in BP with the WHQ and the WHOQoL-bref. A stronger correlation was observed with the WHQ. This was expected because the WHQ was also developed for this specific population, whereas the WHOQoL-bref is a generic instrument; the inclusion of the WHOQoL-bref sought to identify general changes in the QoL not specifically related to the climacteric syndrome.

When evaluating the discriminant validity of the BP/CS, we observed significantly different scores between groups with different levels of schooling and family income, presence or absence of vasomotor symptoms (hot flushes), and frequency of physical activity. Concerning schooling, we observed that higher schooling was associated with more QoL. Similarly, we observed that higher family income was also associated with better QoL, with no differences in the vasomotor symptoms subdomain.

In our study, the presence of vasomotor symptoms and lack of physical activity were associated with low QoL. This association was not observed in the sexuality and couple relationship domains.

 Comparing age (45–54 years and 55–64 years) or climacteric period (premenopausal and menopausal women with and without HT use) subgroups did not reveal statistically significant differences in the global score and in most areas of the BP/CS, indicating a similar level of QoL. This finding might be explained by the inclusion of women seeking medical care in the presence of symptoms or women already receiving treatment. It is likely that discriminant validity would be reached for these variables using the BP/CS in a population-based survey with random sampling.

## 6. Conclusions

We observed that most questionnaires with 3 or more unanswered items (i.e., invalid) were filled by women without a partner and/or not sexually active. This warrants adjustments in the CS to contemplate this population and ensure that all climacteric women, irrespective of their marital status and sexual activity, can be evaluated.

One limitation of this study was the use of a convenience sample. Thus, the present results cannot be extrapolated for the general population. However, because the Brazilian Portuguese version of the Cervantes Scale was easy to apply and understand, with good psychometric properties (internal consistency, reliability, and validity), we believe this version can be used to assess QoL in climacteric women in Brazil.

## Figures and Tables

**Table 1:** Demographic characteristics of brazilian women participating in the validation of the brazilian portuguese version of the cervantes scale (*n* = 180).

Variable	
Age (years)	52.3 ± 5.0

Skin color	
White	162 (90.0%)
Mixed	10 (5.6%)
Black	8 (4.4%)

Schooling	
Elementary	94 (52.2%)
High school	40 (22.2%)
University	46 (25.6%)
Professionally active	97 (53.9%)

Monthly family income	
1 to 2 minimum wages	28 (15.6%)
3 to 4 minimum wages	57 (31.7%)
5 to 7 minimum wages	52 (28.9%)
8 to 10 minimum wages	15 (8.3%)
>11 minimum wages	28 (15.6%)

Smoking (cigarettes/day)	
No	153 (85.0%)
Up to 10	12 (6.7%)
10 to 20	14 (7.8%)
>20	1 (0.6%)

Weekly alcohol consumption	
No	129 (71.7%)
1 to 2 times	46 (25.6%)
≥3 times	5 (2.8%)

Physical activity*	
No	89 (49.4%)
1 to 2 times/weeks	42 (23.3%)
≥3 times/weeks	49 (27.2%)
Hot flashes	78 (43.3%)

Clinical status	
Premenopause	83 (46.1%)
Menopause with hormone replacement	46 (25.6%)
Menopause without hormone replacement	51 (28.3%)

Variables expressed as median ± standard deviation or absolute and relative frequency.

*≥30 daily minutes.

**Table 2:** Correlation between the domains and subdomains of the brazilian portuguese version of the cervantes scale.

Domains of the Cervantes Scale	A		B		C		D		E		F		G		Global score
	*r*	*P*	*r*	*P*	*r*	*P*	*r*	*P*	*r*	*P*	*r*	*P*	*r*	*P*	
Menopause and health (A)	1														
Vasomotor symptoms (B)	0.74	<0.001	1												
Health (C)	0.86	<0.001	0.54	<0.001	1										
Aging (D)	0.88	<0.001	0.46	<0.001	0.62	<0.001	1								
Psychic (E)	0.72	<0.001	0.38	<0.001	0.73	<0.001	0.64	<0.001	1						
Sexuality (F)	0.23	0.002	0.06	0.427	0.08	0.278	0.35	<0.001	0.34	<0.001	1				
Couple relationship (G)	0.09	0.214	−0.03	0.652	0.07	0.384	0.15	0.040	0.24	0.001	0.60	<0.001	1		
Global score	0.88	<0.001	0.56	<0.001	0.77	<0.001	0.83	<0.001	0.88	<0.001	0.56	<0.001	0.43	<0.001	1

*r*: correlation coefficient.

**Table 3:** Correlation between the domains and subdomains of the brazilian portuguese versions of the cervantes scale, women's health questionnaire and the WHOQOL-bref.

Cervantes Scale Domains	Women's Health Questionnaire	WHOQoL-bref
Menopause and health	0.71	−0.58
Vasomotor symptoms	0.46	−0.30
Health	0.60	−0.48
Aging	0.67	−0.59
Psychic	0.68	−0.63
Sexuality	0.47	−0.48
Couple relationship	0.29	−0.35
Total Cervantes	0.79	−0.71

*P* < 0.001 for all correlations.

The values express the correlation coefficient.

**Table 4:** Mean cervantes scale scores in brazilian women according to schooling.

Domain/Subdomain	Education			
	Primary (*n* = 94)	Secondary (*n* = 40)	Higher (*n* = 46)	*P*
Menopause and health	27.9 ± 14.0	20.4 ± 13.0	15.9 ± 9.8	<0.001
Vasomotor symptoms	4.7 ± 4.3	3.2 ± 3.9	2.6 ± 3.6	0.008
Health	8.5 ± 5.9	6.6 ± 4.8	6.4 ± 3.9	0.036
Aging	14.6 ± 6.8	10.6 ± 6.5	6.9 ± 4.7	<0.001
Psychic	12.7 ± 10.6	8.2 ± 7.6	5.8 ± 5.1	<0.001
Sexuality	9.5 ± 5.8	7.6 ± 4.4	6.6 ± 4.6	0.004
Couple relationship	4.0 ± 4.4	3.0 ± 3.1	3.0 ± 3.3	0.249
Global score	54.0 ± 26.4	39.2 ± 20.9	31.2 ± 16.0	<0.001

Variables expressed as average ± standard deviation.

**Table 5:** Mean cervantes scale scores in brazilian women with or without hot flashes.

Domain/Subdomain	Hot flashes		
	Missing (*n* = 102)	Present (*n* = 78)	*P*
Menopause and health	17.0 ± 11.2	31.2 ± 12.6	<0.001
Vasomotor symptoms	1.3 ± 1.9	7.2 ± 3.8	<0.001
Health	6.0 ± 4.8	9.7 ± 5.2	<0.001
Aging	9.7 ± 6.6	14.4 ± 6.8	<0.001
Psychic	7.8 ± 8.5	12.7 ± 9.6	<0.001
Sexuality	7.9 ± 5.4	9.0 ± 5.2	0.183
Couple relationship	3.3 ± 4.0	3.7 ± 3.8	0.523
Global score	36.0 ± 23.0	56.6 ± 22.5	<0.001

Variables expressed as average ± standard deviation.
